# High-current density and high-asymmetry MIIM diode based on oxygen-non-stoichiometry controlled homointerface structure for optical rectenna

**DOI:** 10.1038/s41598-019-55898-x

**Published:** 2019-12-23

**Authors:** Daisuke Matsuura, Makoto Shimizu, Hiroo Yugami

**Affiliations:** Department of Mechanical Systems Engineering, Graduate School of Engineering, Tohoku University, Sendai, Japan

**Keywords:** Electronic devices, Electronic and spintronic devices

## Abstract

Optical rectennas are expected to be applied as power sources for energy harvesting because they can convert a wide range of electromagnetic waves, from visible light to infrared. The critical element in these systems is a diode, which can respond to the changes in electrical polarity in the optical frequency. By considering trade-off relationship between current density and asymmetry of IV characteristic, we reveal the efficiency limitations of MIM diodes for the optical rectenna and suggest a novel tunnel diode using a double insulator with an oxygen-non-stoichiometry controlled homointerface structure (MO_x_/MO_x−y_). A double-insulator diode composed of Pt/TiO_2_/TiO_1.4_/Ti, in which a natural oxide layer of TiO_1.4_ is formed by annealing under atmosphere. The diode has as high-current-density of 4.6 × 10^6^ A/m^2^, which is 400 times higher than the theoretical one obtained using Pt/TiO_2_/Ti MIM diodes. In addition, a high-asymmetry of 7.3 is realized simultaneously. These are expected to increase the optical rectenna efficiency by more than 1,000 times, compared to the state-of-the art system. Further, by optimizing the thickness of the double insulator layer, it is demonstrated that this diode can attain a current density of 10^8^ A/m^2^ and asymmetry of 9.0, which are expected to increase the optical rectenna efficiency by 10,000.

## Introduction

Recently, optical rectennas have attracted attention as new photoelectric conversion systems with the potential to surpass conventional photovoltaic systems. The rectenna is a system that generates direct-current electric power by directly rectifying an electromagnetic wave, and it is mainly composed of antennas and diodes. Under monochromatic-microwave incidence, the rectenna system achieves a photoelectric conversion efficiency of 90%^1^. Theoretically, it has been demonstrated that 100% conversion efficiency can be achieved, from the visible to infrared light range^2^. Furthermore, the capturing of photons using the wave nature of light by the optical rectenna system is advantageous because any sensitive wavelength can be realized by appropriate antenna design. The optical rectenna is expected to be applied for energy harvesting by extracting electric power from the far infrared rays present in the environment.

In an optical rectenna, the performance of the diode affects the photoelectric conversion efficiency, considerably. Conventional diodes, such as p-n diodes and Schottky barrier diodes, cannot respond to the ultrafast pole changes with the optical frequency. As ultrafast diodes, tunnel diodes that have metal-insulator-metal (MIM) or metal-insulator-insulator-metal (MIIM) structures are strong candidates, and have been extensively studied^3–9^. Recently, Sharma *et al*. reported the first successful photoelectric conversion of sunlight thorough optical rectification using an MIM diode consisting of multiwalled carbon nanotubes^10^.

**Figure 1:**
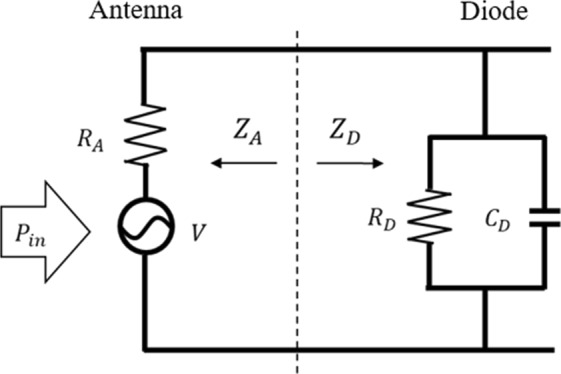
Equivalent circuit of the optical rectenna for calculating *η*_*c*_.

The high-frequency-response efficiency of tunnel diodes is equivalent to the impedance matching efficiency, *η*_*c*_, between the diode and antenna^11^. *η*_*c*_ is defined by calculating the effective power in the diode part assuming an equivalent circuit as shown in Fig. 1, and it is expressed by Eq. (1).

1 $${\eta }_{c}=\frac{4\frac{{R}_{A}{R}_{D}}{{({R}_{A}+{R}_{D})}^{2}}}{1+{\omega }^{2}{C}_{D}^{2}{(\frac{{R}_{A}{R}_{D}}{{R}_{A}+{R}_{D}})}^{2}},$$

where *R*_*A*_[Ω] and *R*_*D*_[Ω] are the resistances of the antenna and diode, respectively, *C*_*D*_[F] is the capacitance of the diode, and *ω*[rad/s] is the angular frequency of the incident wave. *η*_*c*_ includes the diode RC time constant, *τ*[s], which express the response time of the diode. In order to obtain high *η*_*c*_ at the visible-light frequency, *τ*[s] of the diode must be less than 10^−15^ s, i.e., a diode with higher current density has higher *η*_*c*_. The importance of impedance matching has also been mentioned by Sharma *et al*. in ref. ^10^, for improving the total conversion efficiency. For high impedance matching, the Ni/NiO_x_/Ni MIM diodes reported in ref. ^12^ included a high current density of 10^11~12^ A/m^2^ (*τ* < 10^−13^ s) by forming a very thin (~2 nm) and low (0.2 eV) tunnel barrier; however, it remains insufficient for rectifying visible light.

The efficiency, *η*_*β*_, that depicts the rectification performance of the diode is also a crucial parameter. *η*_*β*_ is expressed as the product of the operating voltage, *V*_*D*_, and quantum efficiency, ${\beta }_{i}^{sc}$, as in Eq. (2)^13^.

2 $${\eta }_{\beta }={V}_{D}{\beta }_{i}^{sc}=\frac{{V}_{D}}{{V}_{ph}}[\frac{{I}_{dark}({V}_{D}+{V}_{ph})-2{I}_{dark}({V}_{D})+{I}_{dark}({V}_{D}-{V}_{ph})}{{I}_{dark}({V}_{D}+{V}_{ph})-{I}_{dark}({V}_{D}-{V}_{ph})}],$$

where *V*_*ph*_ is the electric potential equivalent to the photon energy and *I*_*dark*_ is the IV characteristic of the diode in the dark state. When one electron is excited by one photon, the quantum efficiency, ${\beta }_{i}^{sc}$, is the maximum (=1/*V*_*ph*_). Here, *η*_*β*_ becomes unity, when the operating voltage is equal to *V*_*ph*_. In the classical limit, i.e., *V*_*ph*_ → 0, ${\beta }_{i}^{sc}$ corresponds to the classical responsivity, *I*″(*V*)/*I*′(*V*), of the diode. On the other hand, when *V*_*ph*_ is finite, the electron potential is quantized; consequently, ${\beta }_{i}^{sc}$ exhibits a close relationship with the diode asymmetry (the difference between the forward and reverse current at (*V*_*D*_ + *V*_*ph*_) and (*V*_*D*_ − *V*_*ph*_), as in Eq. (2)).

With *η*_*c*_ and *η*_*β*_, the total diode efficiency for the optical rectenna is expressed as in Eq. (3). It indicates that diodes with high current density and high asymmetry are indispensable for achieving high efficiency in the optical rectenna system.

3 $${\eta }_{diode}={\eta }_{c}{\eta }_{\beta }.$$

In this study, we suggest a novel type of MIIM diode, which includes a homointerface structure composed of oxygen-stoichiometric and non-stoichiometric (MO_x_/MO_x−y_) layers as the tunnel layer, for realizing both high asymmetry and high current density. By using a non-stoichiometric metal oxide layer, it is possible to design the tunnel barrier height and form MIIM tunnel barriers, which are impossible to realize with conventional material combinations.

## Results

### Evaluation of the diode performance for the optical rectenna

**Figure 2:**
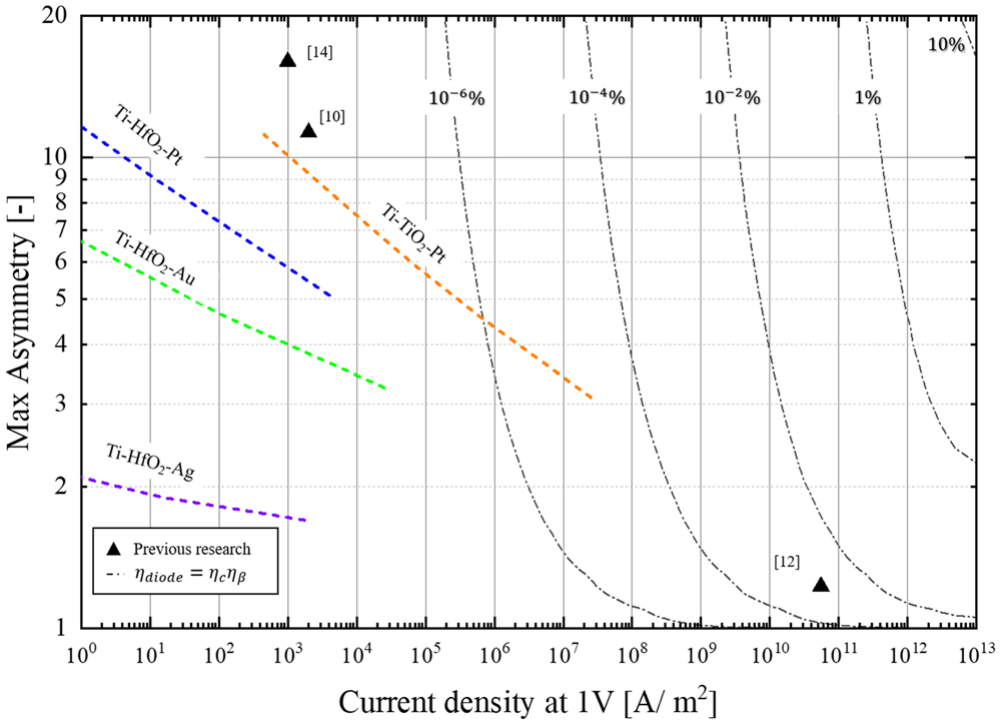
Performance with three tunnel-diodes in previous studies and the theoretical MIM diode performance for each material combination calculated by WKB approximation on varying the barrier thickness from 2–5 nm.

The diode performances for the optical rectenna system can be clearly understood from Fig. 2, which displays the current density at an operating voltage of 1 V on the x-axis and the maximum asymmetry of the diode on the y-axis. For the diode efficiency indicated by the dot-dashed lines in Fig. 2, *η*_*c*_ was calculated from the current density of the diode using Eq. (1), assuming a vacuum impedance *R*_*A*_ = 377 Ω, general tunnel-diode capacitance *C*_*D*_ = 0.01 F/m^2^, and *ω* = 2*π* × 10^14^ rad/s. *η*_*β*_ was calculated using Eq. (2). Here, the diode IV characteristics were approximated with the following simple formulae: ${I}_{forward}(V)=Asymmetry\times {e}^{\alpha {V}_{D}}$ in the forward direction and ${I}_{reverse}(V)=-\,{e}^{-\alpha {V}_{D}}$ in the reverse direction. The constant, *α*, was defined considering an IV curve comparable to that of the conventional MIM diode. Since these efficiency can be obtained with approximately 50 nm square area diode, it is possible to fabricate and realize the efficiency if diode can achieve these current density and asymmetry of I–V characteristic. In Fig. 2, the solid-triangles indicate the diode performance of a previously reported optical rectenna^10,12,14^. The dashed line depicts the theoretical performance of the MIM diode calculated using Eq. (4) for different material combinations^15^.

4 $$J({V}_{D})=\frac{4\pi {m}^{\ast }e}{{h}^{3}}\,{\int }_{0}^{\infty }\,T({E}_{x})d{E}_{x}{\int }_{{E}_{x}}^{\infty }\,\{{f}_{L}(E)-{f}_{R}(E+e{V}_{D})\}dE,$$

where m^*^ is the effective mass of the electron, *e* is the elemental charge, and *h* is the Planck’s constant; *f*(*E*) is the Fermi-Dirac distribution at an energy level, *E*; Suffixes L and R indicate the right and left metals in the tunnel barrier; *T*(*E*) is the transmission probability. The details of the calculation and material parameters are presented in the supporting information. The performance lines were obtained by fixing the barrier height and relative permittivity, and varying the tunnel barrier thickness from 2–5 nm. The effective mass of the electrons was determined using the relationship between the film thicknesses of the insulator, mentioned in a previous research^4^. According to Fig. 2, the Ti/TiO_2_/Pt MIM diode has the best performance among the other MIM diode combinations. This is because of the large work function difference between Ti and Pt, contributing to high asymmetry. In addition, the small tunnel barrier derived from the large electron affinity of TiO_2_ contributes to high current density. However, Fig. 2 also reveals the performance limitation of the MIM diode for the optical rectenna.

### Oxygen-non-stoichiometry-controlled MIIM diode

**Figure 3:**
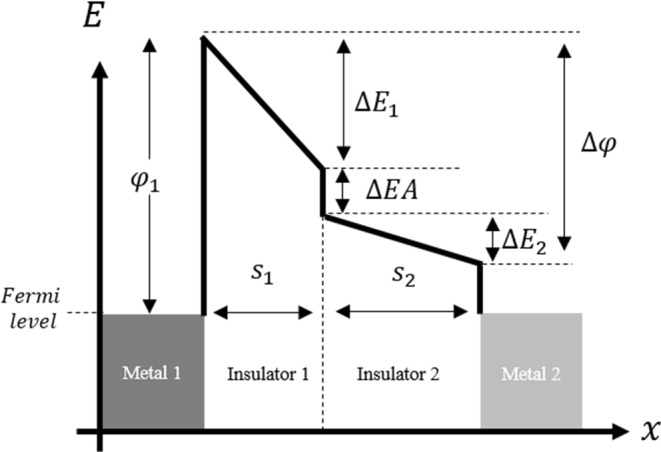
Schematic of the MIIM tunnel barrier. *φ*_1_ is the value obtained by subtracting the electron affinity of the dielectric from the work function of metal-1. *Δφ* is the work function difference between metal-1 and metal-2. *S* is the thickness of the insulator. *ΔEA* is the electron-affinity difference between insulator-1 and the insulator-2.

The MIIM tunnel diode with a double insulator is expected to be a strong candidate for achieving high current density and high asymmetry, simultaneously. Figure 3 shows the energy band diagram of the MIIM diode. With *ε*_1_ and *ε*_2_ as the respective dielectric constants of the insulators, Δ*E*_1_ and Δ*E*_2_ can be expressed by Eq. (5). In order to obtain high asymmetry in the MIIM structure, a difference in the electron affinity between insulator-1 and insulator-2 (Δ*EA*) is needed, as shown in Fig. 3. When a forward voltage is applied, insulator-1 alone behaves as a single tunnel barrier; on the other hand, when a reverse voltage is applied, both insulators function as tunnel barriers, leading to high asymmetry.

5 $$\varDelta {E}_{1}=\frac{{S}_{1}{\varepsilon }_{2}}{{S}_{1}{\varepsilon }_{2}+{S}_{2}{\varepsilon }_{1}}\varDelta {\varphi };\,\varDelta {E}_{2}=\frac{{S}_{2}{\varepsilon }_{1}}{{S}_{1}{\varepsilon }_{2}+{S}_{2}{\varepsilon }_{1}}\varDelta {\varphi }.$$

Here, a combination of Pt/TiO_2_/TiO_2−x_/Ti materials was selected for the MIIM diodes. By incorporating the MIIM structure into Pt/TiO_2_/Ti, which is a material combination that provides the best MIM diode performance, high efficiency is expected. Generally, insulators that exhibit a larger barrier than TiO_2_, such as HfO_2_, Al_2_O_3,_ and SiO_2_, are applied on the Pt side to form MIIM diodes because the electron affinity of TiO_2_ is very high among the other insulators (3.9 eV)^4^ and close to the Ti work function (4.3 eV)^16^. However, this renders it difficult to obtain large current density, although high asymmetry is achieved. Therefore, on the Ti side, TiO_2−x_ that has defect levels within the bandgap of TiO_2_^17^ was applied. The effective conduction level drops to the defect level, and TiO_2−x_ has a larger electron affinity than TiO_2_. Consequently, small-barrier-height MIIM diodes with both high current density and high asymmetry can be obtained.

**Figure 4:**
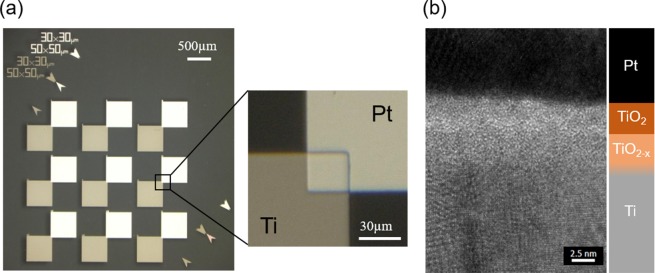
(**a**) Overall view of the diode IV measurement cell and magnified view of the diode portion; nine diodes with a contact area of 900 μm^2^ were fabricated on a silica substrate, and (**b**) Cross sectional view of the diode part, obtained by TEM.

The fabricated Pt/TiO_2_/TiO_2−x_/Ti MIIM diode is shown in Fig. 4. In the fabrication, we use atomic layer deposition method to form the tunnel layer. Therefore, it is considered that the diode is manufactured with high uniformity. Nine diodes were fabricated on a silica substrate, as shown in Fig. 4(a). The diodes were fabricated in the corner of the metal pads, where Pt and Ti overlap for an area of 900 um^2^, as depicted in the magnified image. The cross-section obtained using a transmission electron microscope (TEM) clearly shows the contrast of the upper oxygen-stoichiometric and lower non-stoichiometric oxide layers. The density of each oxide layer was evaluated by the X-ray reflection (XRR) method; the detailed results are presented in the supporting information. From the density analysis, the oxide defects of TiO_2−x_ in the region up to 2 nm from the interface between TiO_2_ and TiO_2−x_ were estimated to be approximately 0.6 (TiO_1.4_). This result indicates that a double insulator possessing two different tunnel barrier heights was fabricated, as expected.

**Figure 5:**
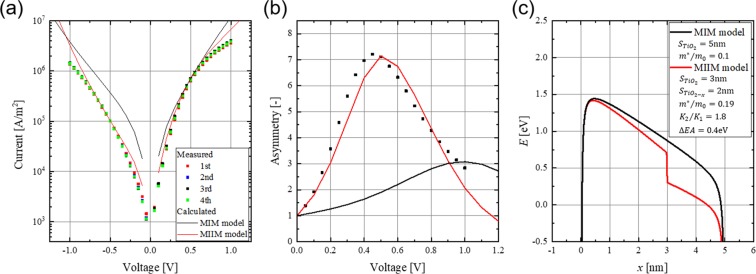
(**a**) IV characteristic and (**b**) asymmetry change with the operating voltage, and (**c**) Energy band diagram of the tunnel barrier used in the calculation. In (**a**,**b**) the plots show the measured IV characteristics; the calculated IV characteristics and asymmetry change using the MIM and MIIM tunnel barrier models shown in (**c**) are depicted by black and red solid lines, respectively.

The evaluation results of the change in the IV characteristics and asymmetry with the operating voltage are displayed in Fig. 5. The IV characteristics of the other diodes fabricated on the same substrate are shown in Fig. S3. Since they show the same characteristics, it can be also an evidence that the fabricated tunnel layer is considered to be uniform. What is concerned about this structure is that the non-stoichiometry changes with voltage application, and the diode characteristics change greatly as the tunnel barrier shape changes. In a previous study, it was reported that the resistance of the HfO_2_ layer, which contained oxygen defects, varied discontinuously, when the oxygen defects were moved by an external bias to create a new conduction path^18^. Oxygen-non-stoichiometry changes are caused by the movement of oxygen vacancies and are relatively fast processes. In order to confirm this, IV measurements were repeated four times. However, no discontinuous change in the resistance was observed in our experiments. Therefore, it can be concluded that the oxygen defects in TiO_2−x_ were sufficiently stable for application in the optical rectenna. The asymmetry of the diode current calculated from the IV characteristics is plotted in Fig. 5(b). On increasing the applied voltage, the asymmetry gradually increased, and reached a maximum value of 7.26 at 0.45 V. In the MIM model, we set *φ*_1_ = 1.7 eV, Δ*φ* = 1.3 eV, and ${\varepsilon }_{Ti{O}_{2}}=18$ as in ref. ^4^. ${S}_{Ti{O}_{2}}$ and m^*^ were varied to fit the measured current density at a forward voltage of 0.5 V. In the MIIM model, we used the same parameters values of *φ*_1_ = 1.7 eV and Δ*φ* = 1.3 eV, and fixed the thicknesses of the oxide layers as ${S}_{Ti{O}_{2}}$ = 3 nm and ${S}_{Ti{O}_{2-x}}$ = 2 nm, respectively, as demonstrated by TEM image in Fig. 4(c). The density calculation result by the XRR method are shown in Supplementary Fig. S4. *ΔEA*, ${\varepsilon }_{Ti{O}_{2-x}}/{\varepsilon }_{Ti{O}_{2}}$, and m^*^ were varied to fit the measured results. Figure 5(c) displays the energy band diagram of the MIM and the MIIM models used for the calculation of the IV characteristics. These results clearly demonstrate that the IV characteristics of the diode fabricated in this study are better matched by the MIIM model, which estimates the thickness parameter obtained by TEM and the XRR method better than in the MIM model. This establishes that the oxygen-non-stoichiometric layer, TiO_2−x_, influences the diode performance as the second insulator layer.

## Discussion

The plot of the diode current density versus the asymmetry clearly establishes the potential of the fabricated diode for application in optical rectennas. Figure 2 reveals that it is difficult to realize high efficiency optical rectennas using conventional MIM diodes, although they are considered as candidates for high-frequency applications. As indicated by the dashed lines that show the calculated performance of the MIM diodes, the current density and the asymmetry are in a trade-off relationship because a small barrier height and large work function difference cannot be obtained between two metals. Therefore, to attain high-efficiency, this relationship must be overcome.

**Figure 6:**
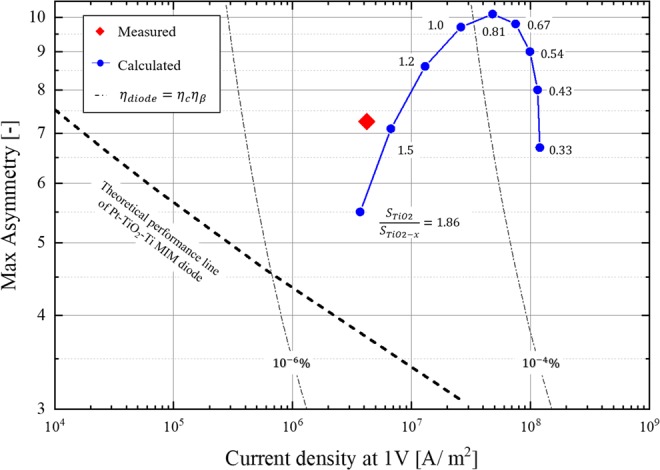
Average performance of the fabricated MIIM diode (red diamond). The blue plot indicates the change in diode performance, when the thickness ratio of TiO_2_ to TiO_2−x_ is varied using the parameters obtained by fitting the measured values with the MIIM model.

The measured results of the fabricated diodes exceed the theoretical ones for the MIM diode, as shown in Fig. 6. The comparison of the measured IV characteristics with the calculated results in Fig. 5 establish that the results using the double insulator model are consistent with the measured ones. Note that the physical properties of the diode materials are not measured or verified by ourselves. However, considering that many other rectenna diode papers show consistency between analytical and measured results using similar physical properties, we think the actual properties used in this study are also not significantly different from the literature data. It is also to be noted that the properties of the non-stoichiometric layer, including the surface defect, gradation of the oxygen defect in the layer, etc., were not considered for the calculations. Supplementary Fig. S4 indicates that the concentration of the oxygen defects in the TiO_2−x_ layer gradually increase, further from the surface. If TiO_2−x_ functions as a semiconductor, the MIM model should be able to explain the IV characteristics. However, as the IV characteristics obtained in this experiment could not be explained by the MIM model, it is expected that TiO_2−x_ behaves as an insulator in the portion, where the oxygen defects are less. In a tunnel barrier with a work function difference between the metals (Fig. 3), an electric field is generated in the dielectric such that the Fermi levels of the two metals coincide. Therefore, the free electrons in TiO_2−x_ are consumed as a true current to cancel the internal electric field, and it is expected that the TiO_2−x_ layer behaves as an insulator, substantially. Even with the simple double insulator model, the calculated IV characteristics match well with the measured ones. Therefore, we can conclude that this IV characteristic is strongly derived from the double insulator effect.

Figure 6 shows the average performance of the fabricated diodes (indicated by the red diamond). The average current density at a forward bias of 1 V is 4.2 × 10^6^ A/m^2^ and the maximum asymmetry at 0.45 V is 7.26. The proposed diode achieves a current density that is nearly 400 times greater, compared to the theoretical one for a Pt/TiO_2_/Ti MIM diode with equivalent asymmetry. The asymmetry is not as good as that reported in a previous study (asymmetry = 16) for photoelectric conversion^10^; however, the current density is greater by 1,000. The blue plot in Fig. 6 depicts the change in diode performance, when the film thickness ratio, ${S}_{Ti{O}_{2}}/{S}_{Ti{O}_{2-x}}$, is varied, while maintaining the total film thickness at 5 nm. For the effective electron affinity and TiO_2−x_ dielectric constant, the value calculated by the fitting in Fig. 5 are used. This analysis reveals that by optimizing the film thickness ratio, it is possible to achieve a current density of approximately 10^8^ A/m^2^ with a maximum asymmetry of nine, which results in an optical rectenna efficiency that is approximately 10,000 times more than that of the state-of-the-art system.

## Methods

Approximately 50-nm of Ti layer was sputtered onto an SiO_2_ substrate in an Ar atmosphere of 0.5 Pa with a sputtering power of 300 W. The anode electrodes are patterned by photolithography and transferred to the Ti layer by fast atom beam (FAB) dry etching; SF_6_ was used as the etching gas. The non-stoichiometric layer (TiO_2−x_) was formed by heating sputtered Ti film at 200 °C, at atmospheric pressure. The stoichiometric layer (TiO_2_) was formed on top of the above layer by the atomic layer deposition (ALD) method. Ti[N(CH_3_)_2_]_4_ was used as the precursor and the stage temperature was 200 °C. Approximately 70-nm Pt was formed by sputtering, under same conditions as those used for Ti. The cathode electrodes were fabricated using same process as that used for the anode.

## Conclusion

In this study, we analytically show that it is difficult to realize a good diode for optical rectenna device only by optimizing the material in a general MIM tunnel diode because they have trade-off relationship between current density and asymmetry of IV characteristic. Therefore, we proposed a novel MIIM diode, which uses a homointerface structure composed of an oxygen-non-stoichiometry-controlled metal oxide tunnel layer for realizing high asymmetry and high current density simultaneously. The performance of the novel MIIM diode was experimentally confirmed by fabricating a Pt/TiO_2_/TiO_2−x_/Ti MIIM diode using the oxygen-non-stoichiometric layer, TiO_2−x_, as the second layer. The fabricated diode achieved a current density that was nearly 400 times greater, compared to the theoretical one in a Pt/TiO_2_/Ti MIM diode with equivalent asymmetry. The total efficiency of the fabricated diode was approximately 1,000 times greater than that in a previous research on the optical rectenna, which achieved the highest efficiency under visible light. The IV characteristics obtained in this study agreed well with the theoretical calculated values fitted by the MIIM model. In addition, the theoretical performance for optimized TiO_2_ and TiO_2−x_ layer thicknesses was calculated, and it was demonstrated that a maximum asymmetry of nine and a current density of approximately 10^8^ A/m^2^ can be achieved. This is expected to increase the optical rectenna efficiency approximately 10,000 times, compared to the state-of-the-art system.

## Supplementary information

Supproting information
