# Supplementary material for: High-current density and high-asymmetry MIIM diode based on oxygen-non-stoichiometry controlled homointerface structure for optical rectenna
Source: Sci Rep. 2019 Dec 23;9:19639. doi: 10.1038/s41598-019-55898-x (PMC6928205; doi:10.1038/s41598-019-55898-x)
Supplement: Supplementary file 1 — Supproting information [file 41598_2019_55898_MOESM1_ESM.docx]

**Supporting information**

**High-current density and high-asymmetry MIIM diode**

**based on oxygen-non-stoichiometry controlled**

**homointerface structure for optical rectenna**

Daisuke Matsuura^1^, Makoto Shimizu^1^, and Hiroo Yugami^1^

^1^Department of Mechanical Systems Engineering, Graduate School of Engineering, Tohoku University

**Diode-IV-characteristics calculation method**

The tunnel current given by Eq. (4) includes the transmission probability, $T(E)$. We applied the WKB approximation in Eq. (S-1) for calculating $T(E)$. $P(E)$ is the Gamow’s permeability factor, expressed by Eq. (S-2). $V(x)$ is a function of the tunnel barrier height with respect to the changes in the *x-*direction.

|  | $T\left( E \right)=\frac{e^{-2P\left( E \right)}}{\left( 1+\frac{e^{-2P\left( E \right)}}{4} \right)^{2}}.$ | (S-1) |
| --- | --- | --- |
|  | $P\left( E \right)=\frac{1}{\hbar}\int_{a}^{b} \sqrt{2m^{*}\left( V\left( x \right)-E \right)}dx.$ | (S-2) |

$V(x)$ of a general MIM diode is depicted in Figure S-1 and expressed by Eq. (S-3).

|  | $V\left( x \right)=\varphi_{1}-\left( \Delta\varphi+eV_{D} \right)\frac{x}{s}-1.15ln \left[ 2 \right]\frac{e^{2}}{8\pi\varepsilon_{1}\varepsilon_{0}}\frac{S}{x\left( x-S \right)},$ | (S-3) |
| --- | --- | --- |

where $\varphi_{1}$ is a value obtained by subtracting the electron affinity of the dielectric from the work function

of metal-1, $\Delta\varphi$ is the work function difference between metal-1 and metal-2, and $S$ is the thickness of

the insulator. The second term of this equation depicts the image force effect, which causes a round-off in

the barrier corners, rendering the barrier narrower and lower. $\varepsilon_{1}$ is the relative dielectric constant of the insulator.

The theoretical IV characteristics of the MIM diode are shown in Fig. S-2, wherein the film thickness was varied from 2–5 nm. In the calculation, the effective electron mass was determined by the relationship between the insulator film thickness, as per previous research [S-1]. As the film thickness increases, the current density decreases and the asymmetry increases. In the calculation of the theoretical performance line shown in Fig. 2, the work functions of Ti, Pt, Mo, and Ir were 4.3 eV, 5.6 eV, 4.6 eV, and 5. 3 eV [S-2], respectively. The electron affinities of TiO_2_ and HfO_2_ were considered to be 3.9 eV [S-1] and 2.5 eV [S-3], respectively.

Fig. S-2. (a) Calculation results of the Pt/TiO_2_/Ti MIM diode performance by varying the film thickness, (b) IV characteristics, and (c) asymmetry.


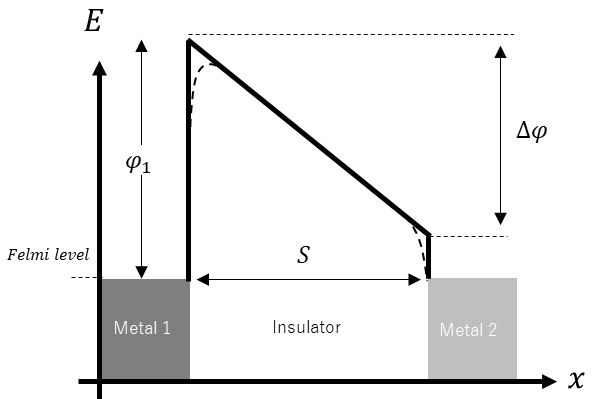


Fig. S-1. Energy band diagram of the MIM tunnel barrier. The solid line indicates the first and second terms of Eq. (S-3), whereas the dashed line represents the barrier considering the third term.

**I-V characteristics of the fabricated diodes**

Measured I-V characteristics of the fabricated diodes in a same substrate are shown below. Since, these five diodes show same I-V characteristics, the diode can be considered to be manufactured with high uniformity.

Fig. S-3 Measured I-V characteristics of diodes fabricated on same substrate.

**Evaluation of the oxide defects (*x*) in the TiO_2-x_ layer**

The oxide defects, *x,* of TiO_2-x_ can be calculated from the film density using Vegard’s rule. For film-density measurements, the X-ray reflection (XRR) method was adopted. In this method, X-ray was incident on the multilayer film at a low angle to obtain the reflection spectrum, which contains information on the film thickness, density, and interface roughness. The density of the film was obtained by fitting the reflection spectrum, calculated from the virtual model of the film, to the measured spectrum. In Fig. S-4 (a), the film density profile across the Pt/TiO_2_/TiO_2-x_/Ti layers, obtained by the XRR method, is converted to the amount of oxide defects, *x*. The fitting results shown in Fig. S-4 (b) indicate that the fitting curve generated from the layered model (Fig. S-4 (a)) is in good agreement with the measured curve. Applying Vegard's rule, *x* = 2 at a density of 3.16 g/cm^2^ for the TiO_2_ layer formed by ALD and *x* = 0 at a density of 4.48 g/cm^2^ for the Ti film. It was demonstrated in previous research that the density of TiO_2_ film formed by ALD was lower than that of the bulk, when the crystal structure was amorphous [S-4]. According to the TEM image shown in Fig. 4(b), the TiO_2_ as well as TiO_2-x_ layers can be considered as amorphous because there is no electron diffraction. From the fitting results, it can be established that the oxygen non-stoichiometric layer exhibits substantially constant oxygen defects up to 2 nm from the surface, and the oxygen deficiency gradually increases from 2–3.5 nm.


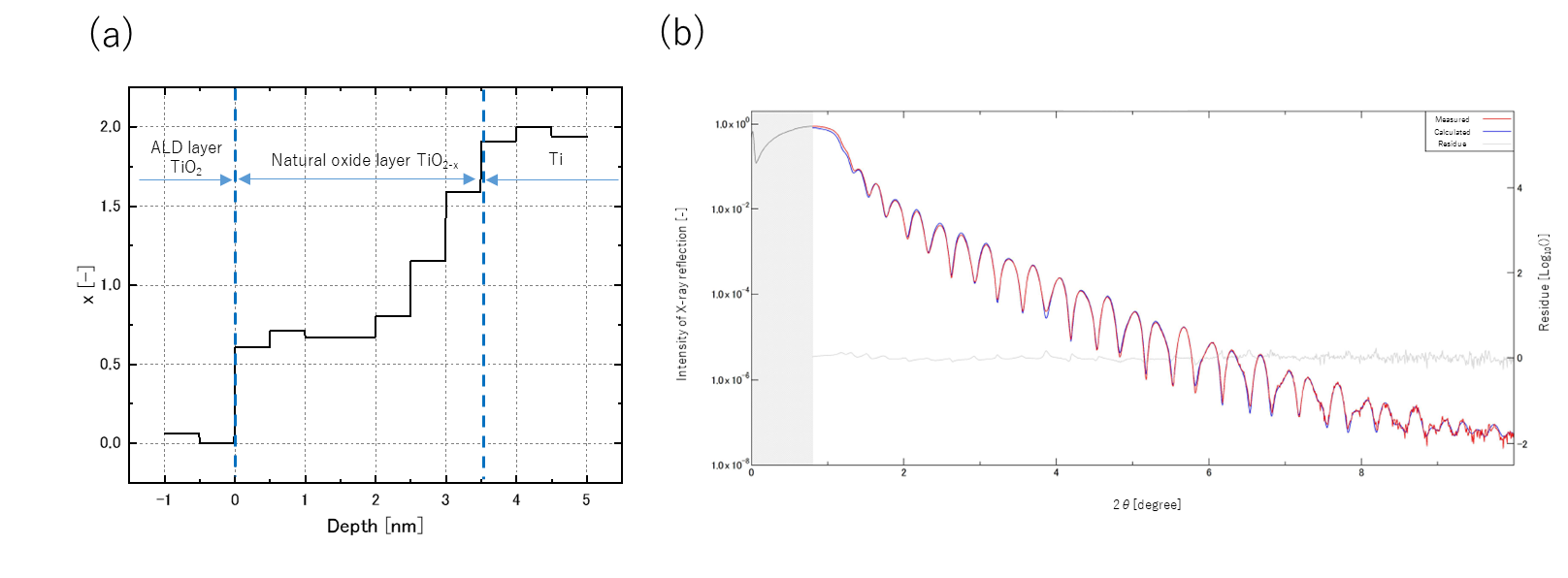


Fig. S-4 (**a**) Distribution of the oxide defects, x, in TiO_2-x_, calculated from the film density, and (**b**) Reflected intensity of the MIIM structure (red line) and calculated value of the reflected intensity for the model utilized to determine the film density (blue line).

**References**

S-1. Cui, Q. S. et al*.* Ultrathin and atomically flat transition-metal oxide: Promising building blocks for metal-insulator electronics. *ACS Appl. Mater. Interfaces* **8**, 34552–34558 (2016).

S-2. Herbert, B. M. The work function of the elements and its periodicity. *J. Appl. Phys.* **48**, 4729, doi:10.1063/1.323539 (1977).

S-3. Alimardani, N. et al*.* Impact of electrode roughness on metal-insulator-metal tunnel diodes with atomic layer deposited Al_2_O_3_ tunnel barriers. *J. Vac. Sci. Technol. A* **30**, 5 (2012).

S-4. Piercy, B. D., Leng, C. Z. & Losego, M. D. Variation in the density, optical polarizabilities, and crystallinity of TiO_2_ thin films deposited via atomic layer deposition from 38 to 150 degrees C using the titanium tetrachloride-water reaction. *J. Vac. Sci. Technol. A* **35**, 5, doi:10.1116/1.4979047 (2017).
